# Trained Immunity Provides Long-Term Protection against Bacterial Infections in Channel Catfish

**DOI:** 10.3390/pathogens11101140

**Published:** 2022-10-02

**Authors:** Lora Petrie-Hanson, Ann E. (Beth) Peterman

**Affiliations:** Department of Comparative Biomedical Sciences, College of Veterinary Medicine, Mississippi State University, Starkville, MS 39759, USA

**Keywords:** trained immunity, histone modifications, ChIP-seq, beta glucan, *Edwardsiella*, phagocytosis, channel catfish

## Abstract

Beta glucan exposure induced trained immunity in channel catfish that conferred long-term protection against *Edwardsiella ictaluri* and *Edwardsiella piscicida* infections one month post exposure. Flow cytometric analyses demonstrated that isolated macrophages and neutrophils phagocytosed higher amounts of *E. ictaluri* and *E. piscicida*. Beta glucan induced changes in the distribution of histone modifications in the monomethylation and trimethylation of H3K4 and modifications in the acetylation and trimethylation of H3K27. KEGG pathway analyses revealed that these modifications affected expressions of genes controlling phagocytosis, phagosome functions and enhanced immune cell signaling. These analyses correlate the histone modifications with gene functions and to the observed enhanced phagocytosis and to the increased survival following bacterial challenge in channel catfish. These data suggest the chromatin reconfiguration that directs trained immunity as demonstrated in mammals also occurs in channel catfish. Understanding the mechanisms underlying trained immunity can help us design prophylactic and non-antibiotic based therapies and develop broad-based vaccines to limit bacterial disease outbreaks in catfish production.

## 1. Introduction

Catfish aquaculture is a high-density animal production system. The presence of sub-acute disease in the system reduces fish growth, while acute disease outbreaks result in fish death and reduced production. Both types of events greatly impact the economic viability of the operation. Understanding the best ways to manage intensive pond aquaculture is complicated because of the complex interaction of the aquatic environment, pathogens and host physiology. Fish biomass, feed input, metabolic waste and minimal water exchange result in the ‘perfect storm’ for several bacterial diseases. Limited commercial vaccines are available for these diseases, and antibiotic medicated feeds are only effective if fish are actively feeding when the medicated feed is provided. Furthermore, there is the risk of bacteria developing antibiotic resistance. Optimizing the innate defenses of fish is needed to reduce losses to infectious diseases.

The first line of defense against bacterial pathogens in fish is mucus covering the integument and gill tissues. The immediate cellular defense against bacterial infections are the cells of the innate immune system are macrophages, neutrophils, dendritic cells, eosinophils, non-specific cytotoxic cells (NCCs), and natural killer (NK) cells. The initial immune responses orchestrated by innate immune cells determine the progression of the disease when fish are first infected. Trained immunity, well documented in mammals, occurs when the innate immune system develops immune memory. Trained immunity provides less-specific protection than the traditionally recognized lymphocyte-based acquired immune response and results in the ability of specific innate immune cells to respond more effectively to certain pathogens and pathogen associated molecular patterns (PAMPs). Trained immunity is generated by epigenetic changes associated with prior PAMP exposure. It results in increased cellular metabolic functions, epigenetic changes and enhanced functional responses of innate immune cells. These changes are long-term and lead to enhanced responses to a variety of pathogens [1].

While less studied in fish, two research models have demonstrated functional trained immunity in teleosts. The ability of innate immune cells of fish to mediate enhanced protective immunity for one month in the absence of lymphocyte based immunity was shown using *rag1*^−/−^ zebrafish and adoptive cell transfers [2]. Further use of the *rag1*^−/−^ model demonstrated long-term protection one month after oral bacterial exposure [3]. Trained immune mechanisms were demonstrated in carp using in vitro studies with head-kidney derived macrophages [4].

There are several important practical benefits of inducing trained immunity in aqua cultured fish. It is less specific than the lymphocyte based acquired immune system so it can provide long-term enhanced protection against many pathogens. After one training event, the fish can be protected against several diseases. Furthermore, the innate defenses are fully functional during early stages in development and at temperatures that are restrictive to acquired immunity. Therefore, trained immunity has the advantage of providing protection to diseases during stages or conditions when acquired immunity may be less functional. Recent studies show that the benefits of trained immunity can be long-lived because epigenetic changes are induced in myeloid progenitor cells [5,6].

It’s been clearly demonstrated that in mammals, functional changes associated with trained immunity are the result of shifts in transcriptional regulation caused by epigenetic changes to the chromatin [1]. Specific modifications of the histones influence the transcription of the genes in the region of the modified chromatin [7]. The genomic locations of the histone modifications are coordinated and cause broad changes in functional pathways of the innate immune cells and influence the type and magnitude of their responses when a pathogen is encountered. Epigenetic reprogramming and chromatin reconfiguration result in long-term sustained changes in cell physiology and functions [7]. Trained immunity in mice results from epigenetic reprogramming of monocytes and macrophages through acetylation or methylation of specific lysines in the histone-H3 [8,9]. These changes occur at promoter regions H3K4me3 [8,10,11], and H3K27ac [9,10,12], enhancer regions H3K4me1 [10,13] and polychrome repression or gene silencing H3K27me3 [14]. Beta glucan exposure can induce these modifications in mammals, via the dectin-1 receptor [7,8,9]. Beta glucan is also a well-documented immune stimulant in fish (reviewed in [15]), but a dectin-1 homologue has not been identified. Instead, beta-glucan recognition is thought to be mediated by a C-type lectin receptor [16].

This study determined the impact of beta glucan induced training of *Ictalurus punctatus*, channel catfish, anterior kidney leukocytes using three complementary approaches, one month after beta glucan exposure. First, we evaluated fish survival after bacterial challenge. Second, we determined the effect on leukocyte phagocytic functions. Third, we evaluated the mechanistic basis using chromatin immunoprecipitation deep sequencing (ChIP Seq) to examine specific histone targets across the channel catfish genome. The sites of the associated epigenetic reprogramming were evaluated for their likely influence on the expression of genes that have recognized functions in immune pathways.

## 2. Results

**Figure 1:**
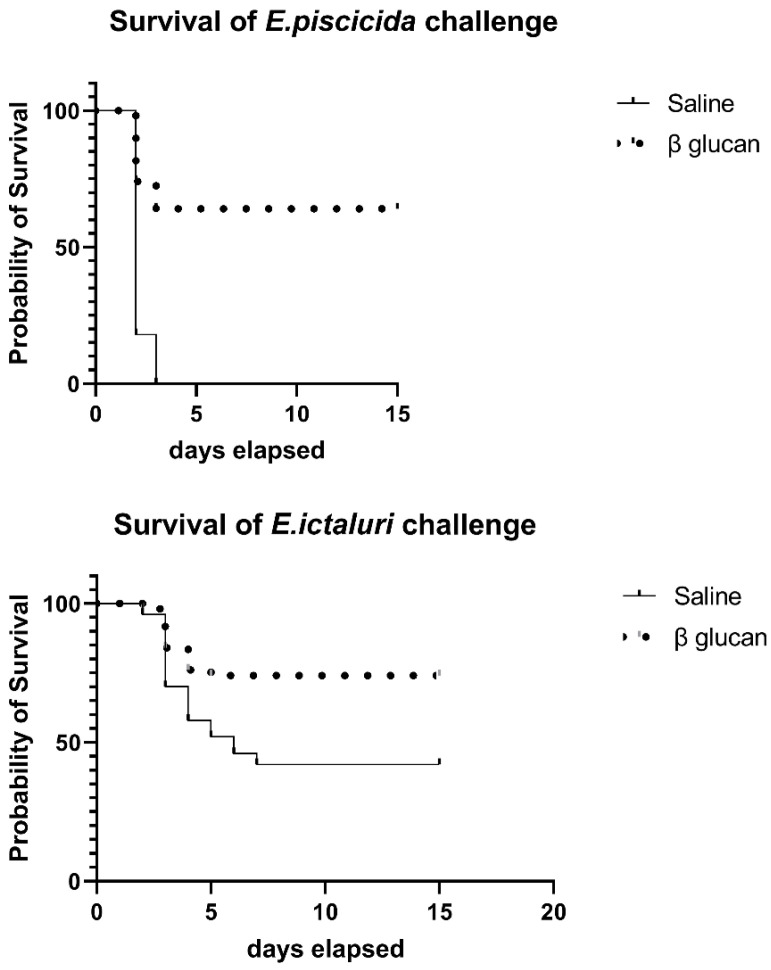
Survival (%) of fish that received an intraperitoneal (IP) saline (0.8 mL/fish) or beta (β) glucan (100 mcg/g of fish in 0.8 mL saline) injection. After 1 month, catfish were challenged by IP *E. piscicida* (3 × 10^4^ CFU/fish). Ten tanks of five fish were used for each treatment. The experiment was repeated for *E. ictaluri* (2.5 × 10^4^ CFU/fish) injection, with ten tanks of five fish used for each treatment. Fish were observed three times per day, moribund fish removed and euthanized and deaths recorded. Statistical analysis was by Kaplan–Meier survival analysis with fifty fish used in each group.

Fish that received an intraperitoneal (IP) injection of beta glucan (bg) followed by an IP injection of *E. piscicida* (bg/*E. piscicida*) 1 month later, demonstrated significantly higher survival (*p* = 0.0001) than IP injected saline controls (saline/*E. piscicida*). Only 36% of the bg/*E. piscicida* injected fish died (64% survival), while 100% of the saline/*E. piscicida* injected fish died. Deaths due to *E. piscicida* infection began at 2 days post injection (dpi) and stopped at 3 dpi (Figure 1). Fish that received an IP injection of bg followed by an IP injection of *E. ictaluri* (bg/*E. ictaluri*) 1 month later, had significantly higher survival (*p* = 0.002) than IP injected saline controls (saline/*E. ictaluri*). The bg/*E. ictaluri* group started dying at 2 dpi and continued to die until 5 dpi. The saline/*E. ictaluri* group began dying at 2 dpi and stopped dying at 7 dpi. Only 26% of the bg/*E. ictaluri* fish died (74% survival), while 58% of saline/*E. ictaluri* fish died (42% survival) (Figure 1).

**Figure 2:**
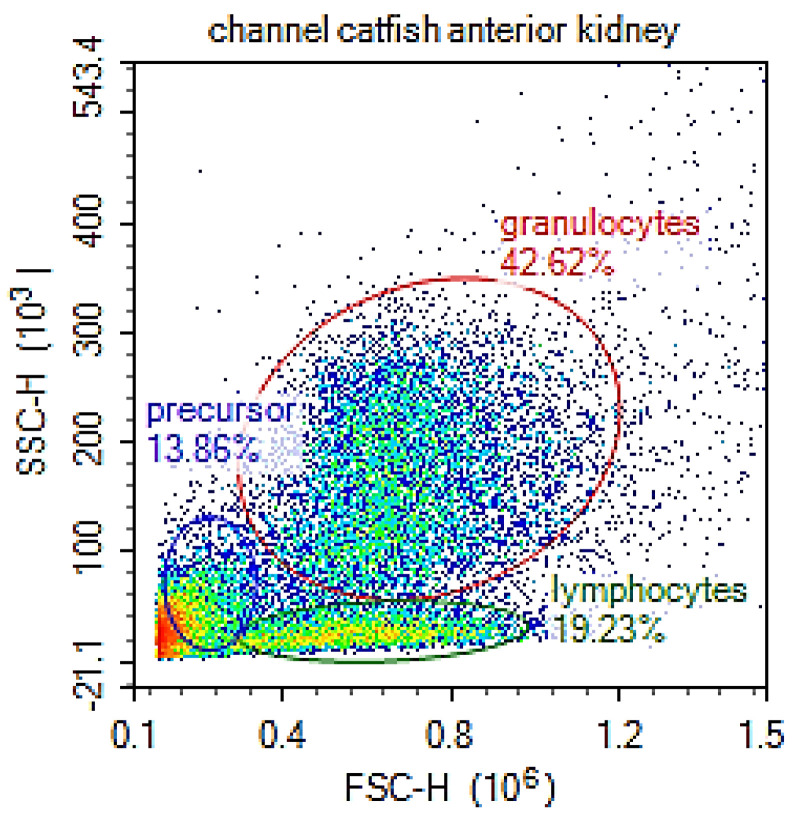
Flow cytometric analysis of channel catfish anterior kidney leukocytes demonstrated different cell populations. On the basis of forward scatter (FSC), or size and side scatter (SSC) or cytoplasmic complexity characteristics and location in a FSC vs. SCC plot, gates were designated granulocytes, lymphocytes and precursors (with thrombocytes).

Flow cytometric examination of isolated anterior kidney (ak) leukocytes demonstrated three general populations (Figure 2). Cells included in gate 1 (granulocyte gate) were granulocytes, macrophages, larger monocytes and NK cells. Cells included in gate 2 (lymphocyte gate) were lymphocytes, small monocytes and some NCCs. Cells included in gate 3 were predominately precursors and thrombocytes. When labeled with antibodies for dendritic cells, macrophages, neutrophils, B cells and IgM, T cells and NK cells and NCCs (Table 1), most dendritic cells, macrophages and neutrophils were in the granulocyte gate. B/IgM+ and T/NK cells were also in the granulocyte gate. B/IgM+ cells, T/NK cells and smaller, less granular NCCs were in the lymphocyte gate. One month after an IP beta glucan exposure, there were significant increases in the number of dendritic cells in the granulocyte gate and significant decreases in the numbers of T/NK cells in the lymphocyte gate (Table 2). When co-incubated with *E. piscicida* transformed with mCherry vector (mCherry: *E*. *piscicida*), neutrophils phagocytosed significantly more mCherry: *E. piscicida* than neutrophils from saline exposed control fish (Table 3). Neutrophils and macrophages from beta glucan exposed catfish phagocytosed significantly more *E. ictaluri* transformed with mCherry vector (mCherry: *E*. *ictaluri*) than neutrophils and macrophages from control fish (Table 4).

### ChIP Sequencing and H3K4 and H3K27 Chromatin Reconfigurations

**Figure 3:**
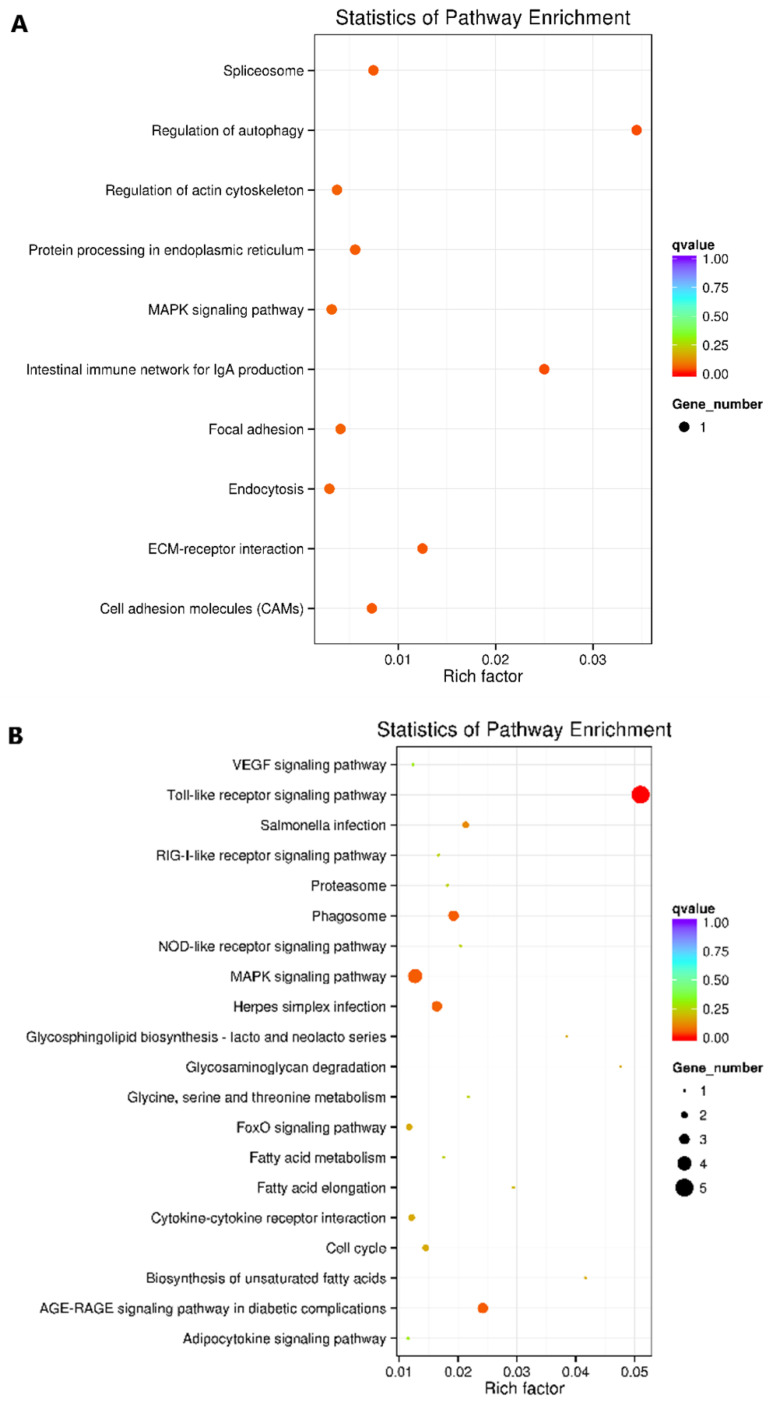

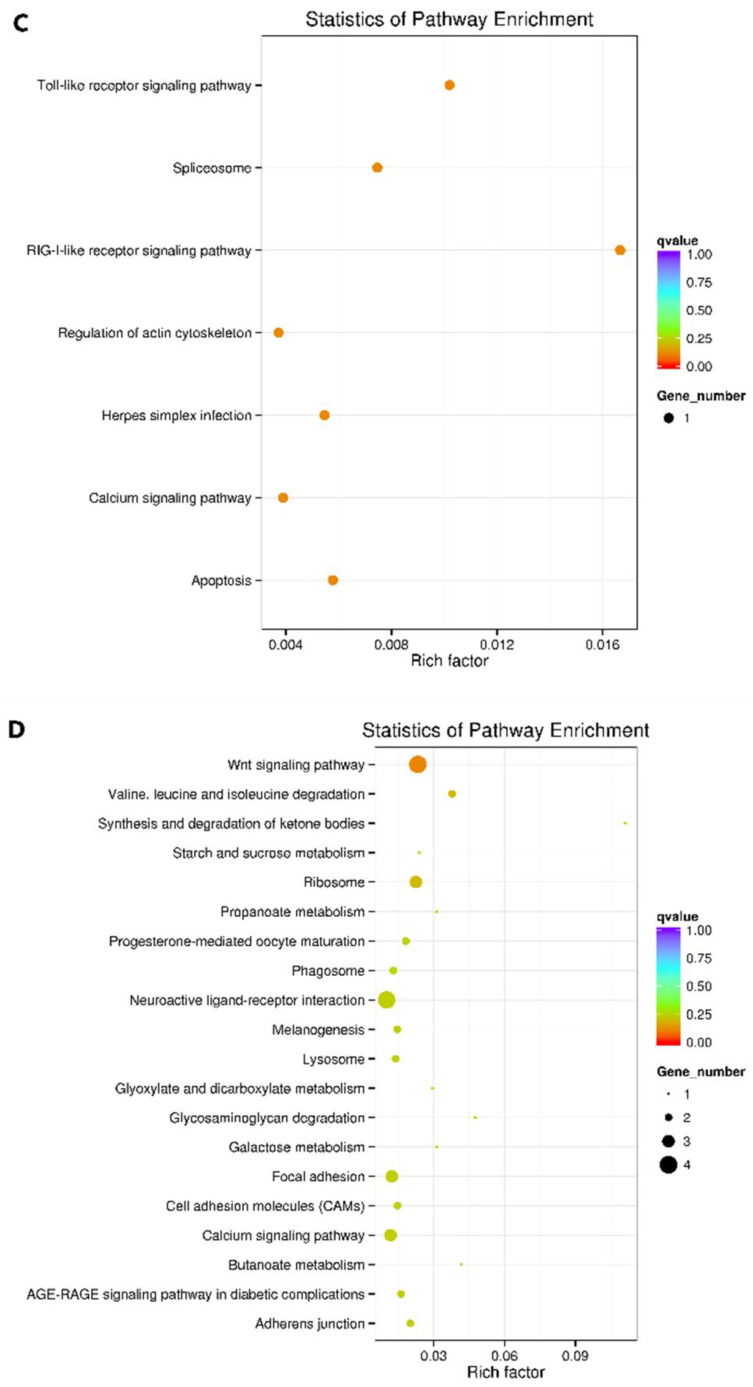
KEGG pathway analyses of H3K4 and H3K27 reconfigurations generated in this study. In these scatter plots, enrichment degree is demonstrated by the rich factor, q value and gene counts enriched to the pathway. Rich factor is the ratio of differentially expressed gene counts to the pathway. The higher the rich factor, the higher the degree of enrichment. The q value ranges from 0 to 1 and is the adjusted *p*-value after multiple hypothesis testing. A low q value indicates greater significance of enrichment. (**A**) Pathways that showed increased H3K4me1 modifications at associated gene loci. (**B**) Pathways that showed increased H3K4me3 modifications at associated gene loci. (**C**) Pathways that showed increased H3K27ac modifications at associated gene loci. (**D**) Pathways that showed decreased H3K27me3 modifications at associated gene loci. The y-axis shows the name of the pathway and the x-axis shows the Rich factor. Dot size represents the number of different genes and the color indicates the q-value.

Each sample provided over 20 million quality reads and over 95% of them mapped to the *I*. *punctatus* genome (Supplemental Table S1). Beta glucan induced modifications of histone 3 at H3K4 and H3K27 in channel catfish. Long-term protection against *E. ictaluri* and *E. piscicida* was associated with histone modifications H3K4me1, H3K4me3, H3K27ac and H3K27me3. Differential occurrence of these modifications identified multiple gene loci that were affected (Supplemental Table S2). Pathway analyses of the genes expressed from these loci (Figure 3) determined pathways associated with phagocytosis and phagocytic functions: phagocytosis, endocytosis, cell adhesion molecules and cytoskeletal movements, and signaling pathways that regulate these cellular functions (Table 5) were included. These findings correlated well with our flow cytometric and survival findings.

## 3. Discussion

### 3.1. Beta Glucan Induced Long-Term Survival against Bacterial Pathogens

Trained immunity is mediated by innate immune cells and occurs when a single exposure to an inducing agent provides long-term protection against a secondary unrelated challenge. This process has been well characterized in humans and mice (reviewed in [1]). In this study we demonstrated that channel catfish exposed to a single beta glucan exposure are protected against lethal disease caused by *E. piscicida* or *E. ictaluri* for at least one month. Trained immunity can be induced by multiple PAMPs [7]. The most researched inducers of trained immunity are beta glucan in mammalian macrophages via dectin-1 [8], and the bacillus Calmette-Guerin vaccination via NOD/2 [22].

Both beta glucan and a NOD-specific ligand induced trained immunity characteristics in a carp macrophage derived cell line [4]. Earlier studies suggest the contribution of trained immunity in fish when protection was documented in situations where the acquired immune system was not functional. For example, there are several studies that demonstrate protection induced in catfish fry after bacterial vaccination. Although channel catfish fry do not develop T or B cells until 7 or 10 days post-hatch, respectively [23], they can be protected when vaccinated prehatch, at the eyed stage [24,25] and as fry [26,27]. Although they do not develop orchestrated acquired immunity until 1 month post hatch, and a secondary antibody response greater than control until vaccinated at 1 month post hatch [28], channel catfish fry demonstrated a protective response to bacterial infection when they were exposed to a live bacterial challenge before they developed a functional acquired immune system. This response provided protection for bacterial challenge 4 weeks after primary exposure but was not associated with a measurable antibody titer. This protection was also associated with increased bacterial clearance rates in the posterior kidney. These studies suggested that in fry, innate immune cells mediated later protection [29]. Another indicator of the involvement of trained immunity in fry is the lower specificity than is typical with lymphocyte-based acquired immunity. Vaccination with *E. ictaluri* provided the same protection to subsequent *E. ictaluri* challenge as to subsequent *Yersinia ruckeri* challenge. Furthermore, vaccination with *Y. ruckeri* provided the same protection to subsequent *E. ictaluri* challenge as to subsequent *Y. ruckeri* challenge [29]. Further, teleost innate immune cells were shown to mediate protective immunity in the T and B cell deficient *rag1*^−/−^ zebrafish [2,3].

### 3.2. Flow Cytometry Suggests Macrophages and Neutrophils Are Involved in Beta Glucan Induced Trained Immune Protection

The FSC-SSC scatter plots of channel catfish ak leukocytes demonstrated cell populations comparable to those in zebrafish, carp and salmonids [30,31,32]. The phagocyte gate in Figure 3 included macrophages, dendritic cells, neutrophils, phagocytic B cells, larger granular NCCs and NK cells. The monoclonal antibody C24a predominately labels T cells, but also labeled some cells with a granularity similar to that of neutrophils [19]. We believe that population is NK cells and is included in the C24a phenotype in the granulocyte gate (Table 2). Nonspecific cytotoxic cell receptor-1 (Nccrp-1), labeled with 5C6, included lymphocyte-like cell populations similar to those described in zebrafish [33]. The larger more granular 5C6+ cells were in the granulocyte gate, while the smaller less granular 5C6+ cells were in the lymphocyte gate. Monoclonal antibody 9E1 labels IgM [21]. The 9E1+ cells in the granulocyte gate were probably phagocytic B cells and IgM+ granulocytes due to the presence of Fc receptors [34,35].

Our findings suggest that some of the protection related to trained immunity may be due to changes in the leukocyte populations. We saw a significant increase in the number of dendritic cells in the granulocyte gate and significant decrease in the number of T cells in the lymphocyte gate in the fish sampled one month after an IP beta glucan exposure (Table 2). However we also found that beta glucan exposure significantly increased the relative percent phagocytosis of mCherry: *E. piscicida* by channel catfish neutrophils (Table 3) and significantly increased the relative percent phagocytosis of mCherry: *E. ictaluri* by channel catfish neutrophils and macrophages (Table 4). Increased phagocytosis following secondary heterologous exposure is a functional characteristic of trained immune cells [7]. Beta glucan induced trained immunity associated with macrophage populations in *rag1*^−/−^ zebrafish [3]. Beta glucan induced increased phagocytosis in primary carp macrophage cultures [4]. In another beta glucan study, isolated ak phagocytes demonstrated enhanced phagocytic and bactericidal abilities following IP injection, and were correlated to lower mortality [36]. Also in studies with *E. ictaluri* vaccinated catfish, isolated macrophages from vaccinated fish phagocytosed more *E. ictaluri* than macrophages from naïve fish [37]. Furthermore, macrophages from the same vaccinated fish produced significantly higher amounts of ROS and NOS [37], which is another functional characteristic of trained macrophages [7]. Though much of the previous research focused on cells of the macrophage linage, our observation of enhanced neutrophil function has also been documented in mammals with trained immunity [38,39].

### 3.3. ChIP Histone Analyses and KEGG Pathway Analyses

After trained immunity has been induced, a cell can function differently upon exposure to a pathogen. An important way to modify immune pathways is by changing transcription regulation, and in mammals with trained immunity, transcriptional change is directed by epigenetic mechanisms. Using ChIP seq, we demonstrated that beta glucan induced the epigenetic modifications H3K4me1, H3K4me3, H4K27ac and H3K27me3 at immune relevant gene locations in channel catfish ak leukocytes. Our findings support our hypothesis that long-term protection induced by beta glucan in fish has the same basis as seen in innate immune training in mammals. The same modifications of histone 3 were present in immune relevant gene locations in myeloid cells of humans and mice after induction of trained immunity [8,9,40]. The H3K27ac, H3K4me1 and H3K4me3 modifications were involved in the enhanced immune response in beta glucan-trained murine macrophages [10], while H3K4me3 and H3K27me3 histone modifications are associated with epigenetic regulation of human immune related genes [13]. In the current study, increased modifications of H3H3K4me1, H3K4me3 and H3K27ac and decreased modifications of H3K27me3 affected multiple pathways associated with bacterial phagocytosis by channel catfish myeloid cells.

KEGG pathway analyses revealed that histone changes upregulated pathways associated with increased phagocytosis, increased endocytosis, increased cell adhesion molecules and increased cytoskeletal movements (Figure 3). H3K27me3 resulted in downregulation of the suppression of the Wnt signaling pathway, ultimately upregulating the Wnt pathway. Changes in these pathways likely increased fish survival and increased bacterial phagocytosis by macrophages and neutrophils.

Phagocytosis is a fundamental mechanism in many eukaryotic cells and is essential to innate and adaptive immune responses. Many proteins and signaling pathways are involved in the phagocytic process. This process includes particle recognition, rearrangement of the cellular cytoskeleton, membrane protrusion around the particle, particle engulfment, phagosome formation and digestion of the particle in the phagolysosome. Intracellular signaling molecules coordinate these processes. There are many different phagocytic receptors and signaling pathways and they have not been fully characterized in catfish leukocytes.

Target recognition involves cell adhesion molecules, extracellular matrix and focal adhesion. Focal adhesions are large, dynamic protein complexes through which the cytoskeleton of a cell connects to the extracellular matrix (ECM). Integrin alpha 4 gene was a highly upregulated component in all of these processes. The actin cytoskeleton pathway mediates and drives phagocytosis, regardless of the organism or the pathogen involved [41]. In combination with the other parts of the cytoskeleton, including intermediate filaments and microtubules, the actin cytoskeleton is responsible for mediating various important cellular processes such as cell structural support, axonal growth, cell migration, organelle transport and phagocytosis. Within this pathway, the integrin alpha 4 gene was highly upregulated. In the endocytosis pathway, heat shock protein 70 was highly upregulated. This protein has been shown to increase the rate and capacity of phagocytosis by six times the basal rate in murine macrophages [42]. In the phagosome pathway, rac1a, colec11, and DYNCH1H1 genes were significantly upregulated (q value < 0.00). Rac1a is involved in phagosome closure [43,44]. Colec11 is a C-type lectin that recognizes carbohydrate antigens and acts in host-defense [45] and has also been shown to modulate phagocytosis and cytokine production in retinal epithelial cells [46]. MAPK pathways include several cell signaling pathways. Map3k7 and Map3k8 genes were highly upregulated and were essential for macrophage bacterial killing [47,48], respectively. The tgf beta2 gene is associated with increased phagocytic activity in macrophages [49] and was highly upregulated in this pathway. The AGE-RAGE pathway recognizes damage associated molecular patterns (DAMPs) and cross talks with the Toll-like receptor (TLR) signaling pathway [50]. The TLR signaling pathway was also highly upregulated after bacterial exposure in beta glucan trained catfish. In the TLR signaling pathway, the TLR9 gene was highly upregulated. This gene has been associated with promoting bacterial phagocytosis [51], and macrophage differentiation into a long-lived phagocytic cell [52]. The calcium signaling pathway was highly upregulated and calcium was one of the first recognized molecules involved in phagocytosis signaling [53]. The RIG-1 signaling pathway was highly upregulated. RIG-1 is involved in anti-viral responses but has recently been shown to also be involved in anti-bacterial responses, specifically in TLR-stimulated phagocytosis [54]. The histone modification H3K27me3 downregulates genes. Downregulation of the Wnt signaling pathway was significantly downregulated, resulting in its upregulation. Wnt signaling regulates and increases macrophage phagocytosis to enhance disease resistance [55]. There are many other pathways that contributed to the increased survival we observed in this study.

## 4. Materials and Methods

### 4.1. Fish Acclimation and Beta Glucan Exposure

**Figure 4:**
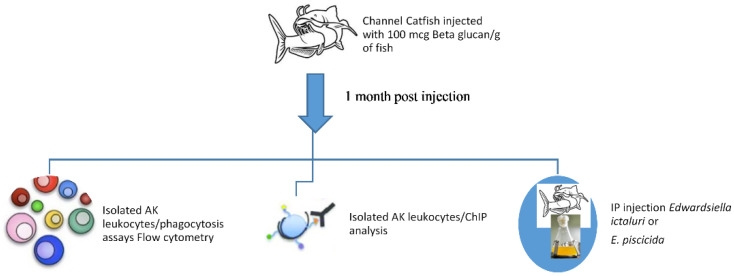
Channel catfish were intraperitoneally injected with saline or beta glucan. After 1 month, studies were performed to determine the effects of beta glucan on the in vivo, cellular and genomic immune functions of channel catfish.

Channel catfish (average weight of 80 g) were held in 30 L tanks, 5 fish per tank. Tanks were supplied with de-chlorinated city water at a flow rate of approximately 0.2 L/min, constant aeration and a water temperature of 28 ± 0.2 °C. Fish were fed a 32% protein commercial catfish feed at 3% of the total fish weight per tank per day. Experimental protocols were approved by the MSU Institutional Animal Care and Use Committee (MSU IACUC). After 1 week acclimation, fish were IP injected with saline (0.8 mL physiological saline/fish) or beta 1–3 glucan linear structure (Calbiochem, CAS 9012-72-0, derived from *Saccharomyces cerevisiae*) (100 mcg/g BW in 0.8 mL physiological saline) by intraperitoneal (IP) injection. Fish were held in this system for 1 month, after which functional and mechanistic studies were performed (Figure 4).

### 4.2. Tank Survival Challenge

*Edwardsiella piscicida* field isolate S11-285 [56] and *Edwardsiella ictaluri* field isolate 2003C [57] were prepared following routine procedures in our lab [3,57]. Fish were challenged by IP injection of *E. piscicida* (3 × 10^4^ CFU/fish) or *E. ictaluri* (2.5 × 10^4^ CFU/fish). Ten tanks of five fish were used for each treatment: bg/*E. piscicida*, bg/*E. ictaluri*, saline/*E. piscicida*, saline/*E. ictaluri,* five tanks were bg/saline and five tanks were saline/saline. Fish were observed 3 times per day, moribund fish removed and euthanized, and deaths recorded. The posterior kidney tissue from random moribund fish were sampled and cultured on tryptic soy agar supplemented with 5% sheep blood to confirm the presence of *E. piscicida* or *E. ictaluri*.

### 4.3. Flow Cytometric Analysis of Cell Populations after Beta Glucan Exposure

Anterior kidney (ak) leukocytes were isolated following routine procedures in our lab [3,33,58]. Five fish were used per treatment with four technical replicates per biological replicate. Briefly, tissues were removed and collected in ice cold FACS buffer (Hanks balanced salt solution without calcium or magnesium with 0.02% bovine serum albumin. Tissues were held on ice and dissociated with a Teflon homogenizer on a 40 μm strainer in FACS buffer. Filtered cells were layered on a Histopaque 1119 gradient (Sigma-Aldrich, Saint Louis, MO, USA, 11191). The suspension was centrifuged at 700× *g* for 20 min. The Buffy layer at the interface between the cell suspension and the gradient was collected, washed with 500 μL FACS buffer and resuspended to a concentration of 1 × 10^6^ cells/mL in FACS buffer.

After washing isolated cells in FACS buffer, 1 × 10^5^ cells/mL were transferred to individual 5 mL flow cytometry tubes in 100 μL aliquots. Cells were fixed with 50 μL 4% paraformaldehyde for 15 min on ice, washed 3 times with 500 μL FACS buffer at 500× *g* at 4 °C, then permeabilized with 50 μL 1X BD Perm/wash buffer (BD, 51-2091KZ) for 5 min on ice, washed 3 times with 500 μL FACS buffer at 500× *g* at 4 °C and all supernatant removed. Fc block (Invitrogen, Waltham, MA, USA,14-9161-73) was added according to the manufacturer’s instructions at 20 μL/tube and incubated on ice for 20 min. Cells were labeled with either FITC or PE labeled antibodies described in Table 1 and incubated on ice for 1 h. The cells were washed with 500 μL FACS buffer three times at 500× *g* at 4 °C for 5 min each wash. The cells were then mixed with 50μL of fluor labeled secondary antibody (fluorescein isothiocyanate (FITC), APC-CY7, or phycoerythrin (PE) at a concentration of 10 μg and incubated on ice for 30 min. Following secondary labeling, the cells were washed three times at 500× *g* at 4 °C for 5 min each wash and re-suspended in 200 μL ice cold FACS buffer.

Isotype controls were used as a negative control to help differentiate non-specific background signal from specific antibody signal. Rat IgG2b isotype control (Invitrogen, 02-9288), mouse IgG2b isotype control (Invitrogen, 02-6300) and rabbit IgG polyclonal isotype control (Abcam, ab37415) were used as negative controls as appropriate. The isotype controls for each fluor were stained using the isotype control as the primary antibody for 1 h followed by incubation with specific fluor for 30 min. Samples were incubated on ice until analyzed.

Flow cytometry analyses of ak leukocytes involved forward scatter (FSC) and side scatter (SSC) determinations on a NovoCyte Acea novosampler. FSC represents cell size in diameter and SSC represents cell granularity, or complexity. Twenty thousand cells were collected each sample. Cells were gated in three areas based on cell sizes and granularity. Unstained, fluorescence minus 1 (FMO), and isotype controls were used to set gates and determine positivity. The positive cells were calculated using the percent positive cells minus the number positive for the isotype control. Results are presented as mean number of cells positive for a specific antibody. Novoexpress software was used for the analysis.

### 4.4. Flow Cytometric Analysis of Bacterial Phagocytosis

The bacterial phagocytosis assay was based on studies described by Russo et al. [27] but utilizing flow cytometry similar to the method of Petit et al. [4] and replacing cherry red bacteria for fluorescent microspheres. Briefly, *E. ictaluri* and *E. piscicida* were transformed with the mCherry vector (Clontech, Mountain View, CA, USA) carrying a kanamycin/neomycin resistant marker. Transformed *E. ictaluri* (mCherry: *E. ictaluri*) and transformed *E. piscicida* (mCherry: *E. piscicida*) were identified using the automated software, Sensititre (Trek Diagnostic Systems Inc., Independence, OH, USA) and by PCR using specific primers and probes [59]. Channel catfish were IP injected with saline or beta glucan at the rate of 100 mcg/g of fish. Five fish were used per treatment, with four technical replicates per biological replicate for flow cytometric analysis. One month later, ak leukocytes were isolated as described above. Bacterial phagocytosis was performed by flow cytometry and was measured by the uptake of mCherry: *E. ictaluri* or mCherry: *E. piscicida* by leukocytes labeled with antibodies. Cells were aliquoted at 1 × 10^6^ cells/mL to 6 well tissue culture plates with four wells for technical replicates and 2 wells for plate controls (no bacteria added). MCherry expressing bacteria were prepared in house by calcium chloride transformation [37] and was grown overnight to log phase and added at 1 × 10^6^ cells/mL to wells of cells from saline or bg injected fish and incubated overnight, aliquoted to 5 mL flow cytometry tubes and labeled with antibodies as listed in Table 1 following the cell labeling procedure as described previously for flow cytometry. Novoexpress software was used for analysis. Bacteria phagocytosed by each phenotype was determined by co-labeling of mCherry labeled bacteria and each specific antibody fluor displayed as a two-color distribution plot analyses, using PE-Texas Red for the bacterial fluorescence display and FITC or PE for the antibody display. The percentage of fluorescent cells for each sample was determined as cells displayed in the dual quadrant of the scatter plot using 20,000 events per sample. Background fluorescence was accounted for by using auto fluorescence emitted by control cells. The percent positive cells were calculated using the percent positive cells in the quadrant minus the number positive for the isotype control divided by the total number of cells collected. Percent phagocytosis was determined by dividing the percentage of cells that were dual stained by the percentage of cells that were dual stained plus the cells that stained by antibody alone. Students *t*-test determined was used to determine if percent phagocytosis was increased by beta glucan exposure.

### 4.5. Data Analysis, Statistical Evaluations and Interpretations

Survival curve analyses were performed by the Kaplan-Meier survival plot using GraphPad Prism version 8.00 for Windows, GraphPad Software, La Jolla, CA, USA, www.graphpad.com (accessed on 15 June 2022). The non-parametric statistic tests Gehan-Breslow-Wilcoxon test and Log ranked (Mantel-Cox) test were used to estimate the statistical significance between the survival curves of saline/*E. piscicida*, saline/*E. ictaluri*, bg/*E. piscicida*, bg/*E. ictaluri*.

In flow cytometry analyses, fluorescence was represented as the mean ± st. dev. All assays used four technical replicates per biological replicate. Statistical significance was determined using ANOVA with LSD correction for multiple comparisons as a post hoc test. Statistical significance was accepted at *p* < 0.05. Statistical analyses were performed using SPSS for Windows 15.0 (SPSS Inc., Chicago, IL, USA). For assay variability 5 replicate assays were used to detect a 10% change in phagocytosis at *p* < 0.05 at a power of 0.89 (type II error rate of 0.11). In flow cytometric phagocytosis assays, all assays were performed with four technical replicates. Relative percent phagocytosis was determined for each antibody population, and an unpaired Student *t*-test used to determine significant differences between saline and beta glucan treatments.

### 4.6. Chromatin Immunoprecipitation (ChIP) and Deep Sequencing Analyses of H3K4 and H3K27

Channel catfish were exposed to bg or saline as described above. After 4 weeks, ak tissues were removed and leukocytes isolated as described above by routine methods, with four biological replicates and four technical replicates of each biological replicate. RNA was purified with PureLink^TM^ RNA Mini Kit (Invitrogen 1218018A) and submitted to Novogene Corporation, Sacramento, CA, USA and used in RNA-sequencing. Ribosomal RNA was depleted, and strand specific cDNA library generated using the TruSeq mRNA stranded kit. Libraries were sequenced on the Lumina system and reads mapped to the channel catfish genome. Reads per kilobase of gene length per million reads (RPKM) values [24] was used to compare expression of genes in bg treated cells to those in non-treated cells.

Chromatin immunoprecipitation: For each biological replicate, chromatin immunoprecipitation was performed on 5 × 10^7^ isolated ak leukocytes using the zymo-spin ChIP Kit (Zymoresearch D5210). Briefly, the cells were fixed in 1% formalin for 7 min, lysed and nuclei harvested. The ChIP Kit protocol was followed and chromatin was sheered by sonication to 200–300 bp fragments. Magnetic bead immunoprecipitation was done using anti-H3K4me1 Ab (Diagenode, Cat. No. CS-037-100), anti-H3K4me3 Ab (Diagenode, Cat. No. pAb-003-050), anti-H3K27ac Ab, (Abcam, Cat. No. ab4729, 0.80 mg/mL) or anti-H3K27me3 (Millipore 07-449). Cross linking was reversed, and the DNA eluted. DNA was evaluated for concentration and quality on an Agilent 2100 expert Bioanalyzer and then submitted to Novogene Corporation (Sacramento, CA, USA) for Lumina sequencing with a minimum of 10 million reads. The obtained reads were aligned to the *I punctatus* genome (GCA_001660625.1 assembly) using HISAT2. Peak analysis was performed with the Model-based Analysis of ChIP-Seq (MACS) algorithm [60]. In order to determine if increased phagocytosis and bacterial killing was due to epigenetic changes, Histone 3 trimethylation of lysine 4, histone 3 methylation of lysine 4, histone 3 acetylation of lysine 27 and trimethylation of histone 3 lysine 27 were determined. Epigenomic profiling of H3K4me1, H3K4me3, H3K27ac and H3K27me3 on the channel catfish transcriptome determined which genes were affected. Pathway analysis used Kyoto Encyclopedia of Genes and Genomes or KEGG.

## 5. Conclusions

Beta glucan induced specific epigenetic reprogramming in channel catfish anterior kidney leukocytes, resulting in enhanced macrophage and neutrophil cell signaling and phagocytosis which provided protection for one month against *E. ictaluri* and *E. piscicida* infections in channel catfish. These data are hallmarks of innate immune system memory, or trained immunity. By clearly documenting the induction of trained immunity in catfish, this study can provide the basis for development of trained immunity-based strategies to improve fish survival in aquaculture systems. More detail on the how the epigenetic changes associated with beta glucan induced trained immunity influence the functions of innate immune cells to enhanced disease resistance are needed. This understanding can help us design and optimize prophylactic measures to limit disease outbreaks in catfish production.

## Figures and Tables

**Table 1:** Antibodies and fluors used for fluorescent activated cell sorting.

Antibody	Fluor	Company/Catalog	Specificity	Reference
Polyclonal Mpeg-1 ^1^		Ana Spec AS-55917	Macrophages	[3,17]
Monoclonal L/CD207 ^2^	PE	Biolegend 405307	Dendritic cells	[18]
Monoclonal 51a ^3^			Neutrophils	[19]
Monoclonal 5C6 ^4^	FITC	Invitrogen, MA5-16528	NCCs	[20]
Monoclonal C24a ^5^			T cells, NK cells	[19]
Monoclonal 9E1 ^6^			B cells, IgM	[21]
Secondary	APC	Invitrogen A21039	Mpeg-1 goat anti rabbit IgG	
Secondary	FITC	Invitrogen F2761	51a, C24a, 9E1 goat anti mouse IgG	

^1^ polyclonal against C terminal region of zebrafish MPEG-1, GenBank Accession #NP_9979021. ^2^ monoclonal against CD207 on cell surface and Birbeck granules of Langerhans cells and subsets of dendritic cells. ^3^ monoclonal against leukocyte specific leucine zipper protein. ^4^ monoclonal against Nccrp-1 and CD11b. ^5^ monoclonal against catfish T cell lineage surface marker. ^6^ monoclonal against catfish IgM H.

**Table 2:** A single intraperitoneal injection of beta glucan increased channel catfish ak leukocyte L/CD207, 9E1 and C24a positive phenotypes labeled with an antibody and gated by FSC and SSC.

Antibody	Gate	Saline	Bg	*p*-Value
L/CD207 (dendritic cells)	Granulocytes	2097 ± 186	3966 ± 589	0.02
	Lymphocytes	24 ± 8	73 ± 28	0.2
Mpeg-1 (macrophages)	Granulocytes	3587 ± 616	3919 ± 1030	0.8
	Lymphocytes	14 ± 5	19 ± 4	0.5
51a (neutrophils)	Granulocytes	2651 ± 765	1864 ± 187	0.4
	Lymphocytes	9 ± 4	10 ± 3	0.8
9E1 (B cells, IgM)	Granulocytes	2819 ± 673	2808 ± 874	0.9
	Lymphocytes	670 ± 158	1586 ± 358	0.06
C24a (T cells/NK cells)	Granulocytes	1561 ± 478	2378 ± 134	0.2
	Lymphocytes	337 ± 243	24 ± 6	0.02
5C6 (NCCs)	Granulocytes	4673 ± 751	6807 ± 698	0.07
	Lymphocytes	148 ± 24	166 ± 42	0.7

**Table 3:** Beta glucan exposure significantly increased the relative percent phagocytosis of mCherry: *E. piscicida* by channel catfish neutrophils. mCherry: *E. piscicida* were co-incubated overnight with isolated ak leukocytes from catfish IP injected with saline or beta glucan 1 month prior to the sampling. Flow cytometry determined dual stained cells, or antibody positive cells that were also expressing mCherry and are listed below as the relative percent of cells within a phenotype that are phagocytosing bacteria. Unpaired *t*-tests determined significance. Data is reported as the mean relative percent phagocytosis ± the standard deviation.

Antibody Positive Cells	Saline	Beta Glucan	*p*-Value
5C6 (NCCs)	12.3 ± 1.9	13.6 ± 1.67	0.6
C24a (T cells and NK cells)	22.4 ± 2.4	29.2 ± 4.3	0.2
51a (neutrophils)	16.1 ± 1.7	26 ± 3.4	0.04
Mpeg (macrophages)	15 ± 1.0	22.7 ± 3.6	0.08
9E1 (B cells/IgM)	12.5 ± 3.2	21.1 ± 3.1	0.1
L/CD207 (dendritic cells)	55.1 ± 6.2	62.3 ± 2.0	0.3

**Table 4:** Beta glucan exposure significantly increased the relative percent phagocytosis of mCherry: *E. ictaluri* by channel catfish neutrophils and macrophages. mCherry: *E. ictaluri* were co-incubated overnight with isolated ak leukocytes from catfish IP injected with saline or beta glucan 1 month prior to the sampling. Flow cytometry determined dual stained cells, or antibody positive cells that were also expressing mCherry and are listed below as the relative percent of cells within a phenotype that are phagocytosing bacteria. Unpaired *t*-tests determined significance. Data is reported as the mean relative percent phagocytosis ± the standard deviation.

Antibody Positive Cells	Saline	Beta Glucan	*p*-Value
5C6 (NCCs)	30.4 ± 12.1	37.9 ± 4.3	0.2
C24a (T/NK cells)	35.5 ± 6.7	44.1 ± 16.4	0.3
51a (neutrophils)	43.9 ± 2.4	68.2 ± 1.5	<0.001
Mpeg (macrophages)	41.0 ± 1.9	69 ± 3.2	<0.001
9E1 (B cells/IgM)	48.1 ± 5.3	52.3 ± 1.19	0.4
L/CD207 (dendritic cells)	55.9 ± 2.9	65 ± 1.8	0.08

**Table 5:** KEGG pathway analysis of genes differentially expressed because of histone modifications demonstrated upregulation of phagocytosis, cytoskeletal arrangement and receptor signaling pathways (all pathways significant at *q* < 0.05). These functions were correlated with increased long-term survival in beta glucan exposed channel catfish.

Histone modification	Pathways with phagocytosis related DEGs, 1 month after beta glucan exposure
H3K4me1 (enhancer) Upregulated	Regulation of actin cytoskeleton MAPK signaling Endocytosis Focal adhesion ECM-receptor interaction Cell adhesion molecules (CAMs)
H3K4me3 (promoter) upregulated	Toll-like receptor signaling Phagosome MAPK signaling AGE-RAGE signaling
H3K27ac (promoter) upregulated	Toll-like receptor signaling RIG-1-like receptor signaling Regulation of actin cytoskeleton Calcium signaling pathway
H3K27me3 (polychrome repression) downregulated	Wnt signaling

## Data Availability

All data is available upon reasonable request to the corresponding author.

## Supplementary Materials

The following supporting information can be downloaded at: https://www.mdpi.com/article/10.3390/pathogens11101140/s1, Table S1: The number of reads and the percentage mapped to the genome for each histone modification in individual samples from channel catfish anterior kidney tissues, one month after beta glucan or saline exposure; Table S2: Kegg pathways and represented genes associated with CHIP-seq differential peaks from channel catfish anterior kidney tissues one month after beta glucan or saline exposure.
