# Supplementary material for: Trained Immunity Provides Long-Term Protection against Bacterial Infections in Channel Catfish
Source: Pathogens. 2022 Oct 2;11(10):1140. doi: 10.3390/pathogens11101140 (PMC9607340; doi:10.3390/pathogens11101140)
Supplement: Supplementary file 1 [file pathogens-11-01140-s001.zip › pathogens-1803285-supplementary.pdf]

Supplemental Table S1. The number of reads and the percentage mapped to the genome for each histone modification in individual samples from channel catfish anterior kidney tissues, one month after beta glucan or saline exposure.

Sample*	Reads	Clean_reads	Mapped	Unique_mapped	Dup_Unique_mapped
P_1_AKIC	pair	24880253	6647628(26.72%)	1167091(17.56%)	231545(19.84%)
P_1_AKIC	read1	24880253	24573939(98.77%)	2412656(9.82%)	444286(18.41%)
P_1_AKIC	read2	24880253	24671626(99.16%)	3957444(16.04%)	1133121(28.63%)
B_1_27a	pair	30497333	8448919(27.70%)	1461048(17.29%)	274495(18.79%)
B_1_27a	read1	30497333	30163592(98.91%)	3471851(11.51%)	1072386(30.89%)
B_1_27a	read2	30497333	30237509(99.15%)	4062478(13.44%)	1168937(28.77%)
B_1_27	pair	33897638	9260737(27.32%)	1601070(17.29%)	324252(20.25%)
B_1_27	read1	33897638	33538931(98.94%)	3834114(11.43%)	1203015(31.38%)
B_1_27	read2	33897638	33611110(99.15%)	4379075(13.03%)	1300340(29.69%)
B_1_AKIC	pair	28661933	7592887(26.49%)	1308397(17.23%)	226188(17.29%)
B_1_AKIC	read1	28661933	28356537(98.93%)	3296397(11.62%)	1029702(31.24%)
B_1_AKIC	read2	28661933	28421236(99.16%)	3974073(13.98%)	1225033(30.83%)
B_1_me1	pair	35620773	9206409(25.85%)	1593143(17.30%)	307886(19.33%)
B_1_me1	read1	35620773	35243837(98.94%)	4019974(11.41%)	1238841(30.82%)
B_1_me1	read2	35620773	35321731(99.16%)	4690116(13.28%)	1405044(29.96%)
B_1_me3	pair	31484040	8296080(26.35%)	1437436(17.33%)	280549(19.52%)
B_1_me3	read1	31484040	31150373(98.94%)	3582981(11.50%)	1121255(31.29%)
B_1_me3	read2	31484040	31214863(99.15%)	4038794(12.94%)	1149826(28.47%)
B_2_27a	pair	31949850	8785048(27.50%)	1517843(17.28%)	287713(18.96%)
B_2_27a	read1	31949850	31603582(98.92%)	3585083(11.34%)	1057350(29.49%)
B_2_27a	read2	31949850	31673597(99.14%)	3996708(12.62%)	1052863(26.34%)
B_2_27	pair	30550832	8234148(26.95%)	1421403(17.26%)	265116(18.65%)
B_2_27	read1	30550832	30228168(98.94%)	3508426(11.61%)	1101319(31.39%)
B_2_27	read2	30550832	30288002(99.14%)	3872573(12.79%)	1091223(28.18%)
B_2_AKIC	pair	28769427	7643777(26.57%)	1311879(17.16%)	228219(17.40%)
B_2_AKIC	read1	28769427	28468860(98.96%)	3284315(11.54%)	1009065(30.72%)
B_2_AKIC	read2	28769427	28528390(99.16%)	3808583(13.35%)	1116533(29.32%)
B_2_me1	pair	32184072	8538292(26.53%)	1478406(17.32%)	263849(17.85%)
B_2_me1	read1	32184072	31845831(98.95%)	3543029(11.13%)	962497(27.17%)
B_2_me1	read2	32184072	31901664(99.12%)	3939068(12.35%)	1004180(25.49%)
B_2_me3	pair	31655819	8382273(26.48%)	1452211(17.32%)	284208(19.57%)
B_2_me3	read1	31655819	31323237(98.95%)	3576995(11.42%)	1094361(30.59%)
B_2_me3	read2	31655819	31384184(99.14%)	3903073(12.44%)	1054717(27.02%)
B_3_27a	pair	33239600	8955360(26.94%)	1551922(17.33%)	282759(18.22%)
B_3_27a	read1	33239600	32877349(98.91%)	3752619(11.41%)	1131698(30.16%)
B_3_27a	read2	33239600	32960247(99.16%)	4436971(13.46%)	1284950(28.96%)
B_3_27	pair	35678278	9497503(26.62%)	1646516(17.34%)	311377(18.91%)
B_3_27	read1	35678278	35295962(98.93%)	4058126(11.50%)	1248582(30.77%)
B_3_27	read2	35678278	35368065(99.13%)	4588917(12.97%)	1304299(28.42%)

B_3_AKIC	pair	30305581	7730857(25.51%)	1331611(17.22%)	221778(16.65%)
B_3_AKIC	read1	30305581	29985782(98.94%)	3561691(11.88%)	1189878(33.41%)
B_3_AKIC	read2	30305581	30061020(99.19%)	4506977(14.99%)	1553347(34.47%)
B_3_me1	pair	30602898	8034034(26.25%)	1393843(17.35%)	246969(17.72%)
B_3_me1	read1	30602898	30279628(98.94%)	3327486(10.99%)	869640(26.14%)
B_3_me1	read2	30602898	30332707(99.12%)	3774081(12.44%)	952984(25.25%)
B_3_me3	pair	31329603	7858248(25.08%)	1357349(17.27%)	252616(18.61%)
B_3_me3	read1	31329603	30999138(98.95%)	3596349(11.60%)	1159191(32.23%)
B_3_me3	read2	31329603	31070545(99.17%)	4142040(13.33%)	1264215(30.52%)
B_4_27a	pair	32904424	8890743(27.02%)	1539863(17.32%)	280579(18.22%)
B_4_27a	read1	32904424	32556302(98.94%)	3776234(11.60%)	1177878(31.19%)
B_4_27a	read2	32904424	32627509(99.16%)	4483752(13.74%)	1397038(31.16%)
B_4_27	pair	32709911	8827632(26.99%)	1526064(17.29%)	298476(19.56%)
B_4_27	read1	32709911	32361283(98.93%)	3712579(11.47%)	1157329(31.17%)
B_4_27	read2	32709911	32428952(99.14%)	4236656(13.06%)	1242627(29.33%)
B_4_AKIC	pair	31571226	7953640(25.19%)	1370539(17.23%)	230902(16.85%)
B_4_AKIC	read1	31571226	31248333(98.98%)	3821162(12.23%)	1360875(35.61%)
B_4_AKIC	read2	31571226	31320327(99.21%)	4840672(15.46%)	1780927(36.79%)
B_4_me1	pair	31074236	8187615(26.35%)	1419786(17.34%)	265065(18.67%)
B_4_me1	read1	31074236	30750488(98.96%)	3331068(10.83%)	849267(25.50%)
B_4_me1	read2	31074236	30800162(99.12%)	3744138(12.16%)	935853(25.00%)
B_4_me3	pair	26452938	6697306(25.32%)	1158485(17.30%)	213381(18.42%)
B_4_me3	read1	26452938	26184662(98.99%)	3068437(11.72%)	988491(32.21%)
B_4_me3	read2	26452938	26234135(99.17%)	3472804(13.24%)	1068716(30.77%)
P_1_27a	pair	26990124	6950137(25.75%)	1190344(17.13%)	197588(16.60%)
P_1_27a	read1	26990124	26763724(99.16%)	2909937(10.87%)	765976(26.32%)
P_1_27a	read2	26990124	26771905(99.19%)	3588572(13.40%)	1073541(29.92%)
P_1_27	pair	26978465	7182744(26.62%)	1229035(17.11%)	211553(17.21%)
P_1_27	read1	26978465	26764118(99.21%)	2642481(9.87%)	488190(18.47%)
P_1_27	read2	26978465	26765501(99.21%)	3336611(12.47%)	751676(22.53%)
P_1_me1	pair	28707593	7698110(26.82%)	1313957(17.07%)	226194(17.21%)
P_1_me1	read1	28707593	28364788(98.81%)	2734084(9.64%)	504153(18.44%)
P_1_me1	read2	28707593	28488785(99.24%)	4431875(15.56%)	1240217(27.98%)
P_1_me3	pair	24591607	6476601(26.34%)	1130272(17.45%)	186498(16.50%)
P_1_me3	read1	24591607	24370251(99.10%)	2422228(9.94%)	421883(17.42%)
P_1_me3	read2	24591607	24368856(99.09%)	3191818(13.10%)	709599(22.23%)
P_2_27a	pair	30809475	8138797(26.42%)	1400051(17.20%)	241384(17.24%)
P_2_27a	read1	30809475	30538285(99.12%)	3278487(10.74%)	812326(24.78%)
P_2_27a	read2	30809475	30545588(99.14%)	3757178(12.30%)	966752(25.73%)
P_2_27	pair	21113070	5697239(26.98%)	982338(17.24%)	155889(15.87%)
P_2_27	read1	21113070	20937146(99.17%)	2097572(10.02%)	382936(18.26%)
P_2_27	read2	21113070	20936056(99.16%)	2650743(12.66%)	600978(22.67%)
P_2_AKIC	pair	24711914	6880014(27.84%)	1202300(17.48%)	243214(20.23%)

P_2_AKIC	read1	24711914	24393927(98.71%)	2409783(9.88%)	470598(19.53%)
P_2_AKIC	read2	24711914	24504641(99.16%)	4077862(16.64%)	1249210(30.63%)
P_2_me1	pair	23913187	6511149(27.23%)	1120354(17.21%)	193143(17.24%)
P_2_me1	read1	23913187	23624924(98.79%)	2305165(9.76%)	419204(18.19%)
P_2_me1	read2	23913187	23724647(99.21%)	3622141(15.27%)	1026248(28.33%)
P_2_me3	pair	22025508	6227338(28.27%)	1077805(17.31%)	179266(16.63%)
P_2_me3	read1	22025508	21825295(99.09%)	2220079(10.17%)	453725(20.44%)
P_2_me3	read2	22025508	21825188(99.09%)	3154968(14.46%)	785710(24.90%)
P_3_27a	pair	27539153	7175182(26.05%)	1224880(17.07%)	191529(15.64%)
P_3_27a	read1	27539153	27311476(99.17%)	3056206(11.19%)	888202(29.06%)
P_3_27a	read2	27539153	27325589(99.22%)	3991152(14.61%)	1330934(33.35%)
P_3_27	pair	22085509	5986158(27.10%)	1033599(17.27%)	166053(16.07%)
P_3_27	read1	22085509	21897375(99.15%)	2180606(9.96%)	381178(17.48%)
P_3_27	read2	22085509	21896691(99.15%)	2769216(12.65%)	612073(22.10%)
P_3_AKIC	pair	25418357	6605489(25.99%)	1151910(17.44%)	197824(17.17%)
P_3_AKIC	read1	25418357	25088495(98.70%)	2443326(9.74%)	442056(18.09%)
P_3_AKIC	read2	25418357	25213672(99.19%)	4373048(17.34%)	1246021(28.49%)
P_3_me1	pair	21947220	5853254(26.67%)	1006280(17.19%)	171325(17.03%)
P_3_me1	read1	21947220	21683922(98.80%)	2104486(9.71%)	376277(17.88%)
P_3_me1	read2	21947220	21775751(99.22%)	3456811(15.87%)	1006189(29.11%)
P_3_me3	pair	24440824	6452407(26.40%)	1118985(17.34%)	176078(15.74%)
P_3_me3	read1	24440824	24224604(99.12%)	2405780(9.93%)	424482(17.64%)
P_3_me3	read2	24440824	24226073(99.12%)	3276627(13.53%)	729268(22.26%)
P_4_27a	pair	26398685	7036291(26.65%)	1201942(17.08%)	187111(15.57%)
P_4_27a	read1	26398685	26177921(99.16%)	3054296(11.67%)	979140(32.06%)
P_4_27a	read2	26398685	26193317(99.22%)	4035100(15.41%)	1449499(35.92%)
P_4_27	pair	25620988	6767023(26.41%)	1169622(17.28%)	195985(16.76%)
P_4_27	read1	25620988	25407937(99.17%)	2515114(9.90%)	441295(17.55%)
P_4_27	read2	25620988	25406400(99.16%)	3125779(12.30%)	673212(21.54%)
P_4_AKIC	pair	21769482	5579012(25.63%)	970418(17.39%)	156465(16.12%)
P_4_AKIC	read1	21769482	21491946(98.73%)	2095200(9.75%)	370127(17.67%)
P_4_AKIC	read2	21769482	21595578(99.20%)	3721863(17.23%)	1035302(27.82%)
P_4_me1	pair	27920799	7491773(26.83%)	1284553(17.15%)	218821(17.03%)
P_4_me1	read1	27920799	27592956(98.83%)	2669108(9.67%)	475112(17.80%)
P_4_me1	read2	27920799	27700590(99.21%)	4177286(15.08%)	1123556(26.90%)
P_4_me3	pair	22981812	6197824(26.97%)	1058020(17.07%)	171015(16.16%)
P_4_me3	read1	22981812	22797925(99.20%)	2299127(10.08%)	472193(20.54%)
P_4_me3	read2	22981812	22799268(99.21%)	3194410(14.01%)	822234(25.74%)

*P =saline injected, B=beta glucan injected, number is biological replicate, AKIC=anterior kidney internal control, 27a=H3K27ac, 27=H3K27me3, me1=H3K4me1, and me3=H3K4me3.

Supplemental Table S2. Kegg pathways and represented genes associated with CHIP-seq differential peaks from channel catfish anterior kidney tissues one month after beta glucan or saline exposure.

Kegg pathway Term	ID	P-Value	Genes associated with Diff peak
**H3K4me1***			
Progesterone-mediated oocyte maturation	dre04914	0.0004	ENSDARG00000055470|ENSDARG00000035966|ENSDARG00000059131|ENSDARG00000017294
Ether lipid metabolism	dre00565	0.0081	ENSDARG00000096445|ENSDARG00000059933
Oocyte meiosis	dre04114	0.0093	ENSDARG00000055470|ENSDARG00000059131|ENSDARG00000035966
Cell cycle	dre04110	0.0095	ENSDARG00000055470|ENSDARG00000035655|ENSDARG00000036096
Notch signaling pathway	dre04330	0.0143	ENSDARG00000044062|ENSDARG00000052091
Glycerophospholipid metabolism	dre00564	0.0389	ENSDARG00000096445|ENSDARG00000059933
alpha-Linolenic acid metabolism	dre00592	0.0569	ENSDARG00000096445
Linoleic acid metabolism	dre00591	0.0656	ENSDARG00000096445
Ribosome	dre03010	0.0660	ENSDARG00000075129|ENSDARG00000104011
Ubiquitin mediated proteolysis	dre04120	0.0711	ENSDARG00000055470|ENSDARG00000007885
Regulation of autophagy	dre04140	0.0883	ENSDARG00000099430
Other types of O-glycan biosynthesis	dre00514	0.1078	ENSDARG00000076111
Basal transcription factors	dre03022	0.1187	ENSDARG00000102998
Intestinal immune network for IgA production	dre04672	0.1187	ENSDARG00000103056
Fructose and mannose metabolism	dre00051	0.1241	ENSDARG00000038398

Fatty acid degradation	dre00071	0.1268	ENSDARG00000038618
Adrenergic signaling in cardiomyocytes	dre04261	0.1284	ENSDARG00000018259|ENSDARG00000017294
Arachidonic acid metabolism	dre00590	0.1481	ENSDARG00000096445
Fanconi anemia pathway	dre03460	0.1508	ENSDARG00000007885
Valine, leucine and isoleucine degradation	dre00280	0.1534	ENSDARG00000059503
Hedgehog signaling pathway	dre04340	0.1586	ENSDARG00000061328
Amino sugar and nucleotide sugar metabolism	dre00520	0.1612	ENSDARG00000038398
Fatty acid metabolism	dre01212	0.1638	ENSDARG00000038618
Sphingolipid metabolism	dre00600	0.1715	ENSDARG00000059933
Glycerolipid metabolism	dre00561	0.1715	ENSDARG00000059933
PPAR signaling pathway	dre03320	0.1917	ENSDARG00000038618
ECM-receptor interaction	dre04512	0.2211	ENSDARG00000103056
Inositol phosphate metabolism	dre00562	0.2259	ENSDARG00000076724
RNA degradation	dre03018	0.2330	ENSDARG00000020043
Cardiac muscle contraction	dre04260	0.2586	ENSDARG00000018259
Adherens junction	dre04520	0.2677	ENSDARG00000036096
TGF-beta signaling pathway	dre04350	0.2700	ENSDARG00000036096

Endocytosis	dre04144	0.2835	ENSDARG00000021924|ENSDARG00000036096
Phosphatidylinositol signaling system	dre04070	0.2878	ENSDARG00000076724
AGE-RAGE signaling pathway in diabetic complications	dre04933	0.3201	ENSDARG00000036096
Gap junction	dre04540	0.3264	ENSDARG00000017294
Spliceosome	dre03040	0.3408	ENSDARG00000021924
Cell adhesion molecules (CAMs)	dre04514	0.3469	ENSDARG00000103056
Melanogenesis	dre04916	0.3489	ENSDARG00000017294
FoxO signaling pathway	dre04068	0.4121	ENSDARG00000036096
Wnt signaling pathway	dre04310	0.4140	ENSDARG00000044062
Protein processing in endoplasmic reticulum	dre04141	0.4265	ENSDARG00000021924
Tight junction	dre04530	0.4265	ENSDARG00000017294
Herpes simplex infection	dre05168	0.4336	ENSDARG00000102998
Focal adhesion	dre04510	0.5327	ENSDARG00000103056
Calcium signaling pathway	dre04020	0.5497	ENSDARG00000042521
Metabolic pathways	dre01100	0.5575	ENSDARG00000096445|ENSDARG00000038398|ENSDARG00000076724|ENSDARG00000059933
Regulation of actin cytoskeleton	dre04810	0.5662	ENSDARG00000103056
MAPK signaling pathway	dre04010	0.6240	ENSDARG00000021924

Neuroactive ligand-receptor interaction	dre04080	0.7115	ENSDARG00000110398
**H3K4me3**			
Toll-like receptor signaling pathway	dre04620	0.0005	ENSDARG00000021509|ENSDARG00000103021|ENSDARG00000061710|ENSDARG00000074849|ENSDARG00000044490
AGE-RAGE signaling pathway in diabetic complications	dre04933	0.0088	ENSDARG00000029018|ENSDARG00000100786|ENSDARG00000074849|ENSDARG00000027087
Biosynthesis of unsaturated fatty acids	dre01040	0.0116	ENSDARG00000016038|ENSDARG00000025904
MAPK signaling pathway	dre04010	0.0163	ENSDARG00000017141|ENSDARG00000021509|ENSDARG00000061710|ENSDARG00000074849|ENSDARG00000027087|ENSDARG00000086778
Fatty acid elongation	dre00062	0.0215	ENSDARG00000016038|ENSDARG00000025904
Cytokine-cytokine receptor interaction	dre04060	0.0223	ENSDARG00000038968|ENSDARG00000054771|ENSDARG00000086778|ENSDARG00000027087
Herpes simplex infection	dre05168	0.0307	ENSDARG00000103021|ENSDARG00000021509|ENSDARG00000003151|ENSDARG00000044490
Ether lipid metabolism	dre00565	0.0313	ENSDARG00000017141|ENSDARG00000053381
Fatty acid metabolism	dre01212	0.0532	ENSDARG00000016038|ENSDARG00000025904
Glycosphingolipid biosynthesis - globo series	dre00603	0.0551	ENSDARG00000036155
Glycerolipid metabolism	dre00561	0.0581	ENSDARG00000036155|ENSDARG00000053381
Sphingolipid metabolism	dre00600	0.0581	ENSDARG00000036155|ENSDARG00000053381
Cell cycle	dre04110	0.0596	ENSDARG00000029018|ENSDARG00000003151|ENSDARG00000027087
Phagosome	dre04145	0.0791	ENSDARG00000074849|ENSDARG00000013266|ENSDARG00000113183
VEGF signaling pathway	dre04370	0.0961	ENSDARG00000017141|ENSDARG00000074849

Inositol phosphate metabolism	dre00562	0.0981	ENSDARG00000056690|ENSDARG00000100786
Wnt signaling pathway	dre04310	0.0984	ENSDARG00000100786|ENSDARG00000003151|ENSDARG00000074849
alpha-Linolenic acid metabolism	dre00592	0.1129	ENSDARG00000017141
Salmonella infection	dre05132	0.1223	ENSDARG00000074849|ENSDARG00000113183
Linoleic acid metabolism	dre00591	0.1295	ENSDARG00000017141
Glycosaminoglycan degradation	dre00531	0.1295	ENSDARG00000071699
Glycerophospholipid metabolism	dre00564	0.1328	ENSDARG00000017141|ENSDARG00000053381
TGF-beta signaling pathway	dre04350	0.1371	ENSDARG00000003151|ENSDARG00000027087
Butanoate metabolism	dre00650	0.1458	ENSDARG00000012468
Phosphatidylinositol signaling system	dre04070	0.1544	ENSDARG00000056690|ENSDARG00000100786
Glycosphingolipid biosynthesis - lacto and neolacto series	dre00601	0.1565	ENSDARG00000018971
Pentose and glucuronate interconversions	dre00040	0.1618	ENSDARG00000053405
Endocytosis	dre04144	0.1717	ENSDARG00000101543|ENSDARG00000099240|ENSDARG00000027087|ENSDARG00000099199
Glycosaminoglycan biosynthesis - heparan sulfate / heparin	dre00534	0.1723	ENSDARG00000059616
GnRH signaling pathway	dre04912	0.1878	ENSDARG00000100786|ENSDARG00000017141
Galactose metabolism	dre00052	0.1879	ENSDARG00000036155
Gap junction	dre04540	0.1946	ENSDARG00000100786|ENSDARG00000086778

Other types of O-glycan biosynthesis	dre00514	0.2081	ENSDARG00000022550
Ribosome	dre03010	0.2106	ENSDARG00000101406|ENSDARG00000075129
Cell adhesion molecules (CAMs)	dre04514	0.2175	ENSDARG00000061454|ENSDARG00000098523
Ubiquitin mediated proteolysis	dre04120	0.2244	ENSDARG00000003151|ENSDARG00000099199
Vascular smooth muscle contraction	dre04270	0.2337	ENSDARG00000100786|ENSDARG00000017141
Lysosome	dre04142	0.2337	ENSDARG00000036155|ENSDARG00000071699
Fructose and mannose metabolism	dre00051	0.2375	ENSDARG00000053405
Regulation of actin cytoskeleton	dre04810	0.2445	ENSDARG00000074849|ENSDARG00000086778|ENSDARG00000002956
Glycine, serine and threonine metabolism	dre00260	0.2565	ENSDARG00000058102
NOD-like receptor signaling pathway	dre04621	0.2705	ENSDARG00000021509
Arachidonic acid metabolism	dre00590	0.2796	ENSDARG00000017141
Valine, leucine and isoleucine degradation	dre00280	0.2887	ENSDARG00000012468
FoxO signaling pathway	dre04068	0.2963	ENSDARG00000029018|ENSDARG00000027087
Proteasome	dre03050	0.2976	ENSDARG00000070674
Protein processing in endoplasmic reticulum	dre04141	0.3148	ENSDARG00000003151|ENSDARG00000044972
RIG-I-like receptor signaling pathway	dre04622	0.3194	ENSDARG00000103021
Lysine degradation	dre00310	0.3364	ENSDARG00000036135

Adrenergic signaling in cardiomyocytes	dre04261	0.3628	ENSDARG00000100786|ENSDARG00000070536
Metabolic pathways	dre01100	0.4169	ENSDARG00000056690|ENSDARG00000069852|ENSDARG00000071699|ENSDARG00000058102|ENSDARG00000017141|ENSDARG00000018971|ENSDARG00000100786|ENSDARG00000053381|ENSDARG00000053405
Adipocytokine signaling pathway	dre04920	0.4261	ENSDARG00000067829
Focal adhesion	dre04510	0.4606	ENSDARG00000086778|ENSDARG00000074849
Adherens junction	dre04520	0.4714	ENSDARG00000074849
ErbB signaling pathway	dre04012	0.5100	ENSDARG00000029018
Oocyte meiosis	dre04114	0.5817	ENSDARG00000003151
Melanogenesis	dre04916	0.5843	ENSDARG00000100786
Insulin signaling pathway	dre04910	0.6670	ENSDARG00000067829
Apoptosis	dre04210	0.6670	ENSDARG00000103021
Tight junction	dre04530	0.6794	ENSDARG00000099109
Neuroactive ligand-receptor interaction	dre04080	0.7207	ENSDARG00000017790|ENSDARG00000054771
Calcium signaling pathway	dre04020	0.8045	ENSDARG00000100786
**H3K27ac**			
RIG-I-like receptor signaling pathway	dre04622	0.0273	ENSDARG00000103021
Toll-like receptor signaling pathway	dre04620	0.0440	ENSDARG00000103021

Spliceosome	dre03040	0.0595	ENSDARG00000103845
Apoptosis	dre04210	0.0761	ENSDARG00000103021
Herpes simplex infection	dre05168	0.0803	ENSDARG00000103021
Calcium signaling pathway	dre04020	0.1109	ENSDARG00000002840
Regulation of actin cytoskeleton	dre04810	0.1157	ENSDARG00000006683
**H3K27me3**			
Wnt signaling pathway	dre04310	0.0023	ENSDARG00000100786|ENSDARG00000096549|ENSDARG00000014571|ENSDARG00000015472
Ribosome	dre03010	0.0093	ENSDARG00000075129|ENSDARG00000104011|ENSDARG00000011201
Valine, leucine and isoleucine degradation	dre00280	0.0130	ENSDARG00000060554|ENSDARG00000021220
Synthesis and degradation of ketone bodies	dre00072	0.0309	ENSDARG00000021220
Neuroactive ligand-receptor interaction	dre04080	0.0386	ENSDARG00000100267|ENSDARG00000005522|ENSDARG00000076824|ENSDARG00000061940
Adherens junction	dre04520	0.0410	ENSDARG00000096549|ENSDARG00000014571
Focal adhesion	dre04510	0.0434	ENSDARG00000096549|ENSDARG00000014571|ENSDARG00000104484
Progesterone-mediated oocyte maturation	dre04914	0.0485	ENSDARG00000024746|ENSDARG00000059131

Calcium signaling pathway	dre04020	0.0487	ENSDARG00000100786|ENSDARG00000076824|ENSDARG00000061940
AGE-RAGE signaling pathway in diabetic complications	dre04933	0.0597	ENSDARG00000100786|ENSDARG00000096549
Glycosaminoglycan degradation	dre00531	0.0668	ENSDARG00000071699
Cell adhesion molecules (CAMs)	dre04514	0.0708	ENSDARG00000100562|ENSDARG00000104484
Melanogenesis	dre04916	0.0717	ENSDARG00000100786|ENSDARG00000014571
Butanoate metabolism	dre00650	0.0755	ENSDARG00000021220
Lysosome	dre04142	0.0771	ENSDARG00000008890|ENSDARG00000071699
Phagosome	dre04145	0.0882	ENSDARG00000096549|ENSDARG00000104484
Galactose metabolism	dre00052	0.0985	ENSDARG00000074556
Propanoate metabolism	dre00640	0.0985	ENSDARG00000060554
Glyoxylate and dicarboxylate metabolism	dre00630	0.1042	ENSDARG00000060554
Starch and sucrose metabolism	dre00500	0.1264	ENSDARG00000074556
NOD-like receptor signaling pathway	dre04621	0.1454	ENSDARG00000024746
Hedgehog signaling pathway	dre04340	0.1614	ENSDARG00000061328
Glutathione metabolism	dre00480	0.1614	ENSDARG00000007377
Arginine and proline metabolism	dre00330	0.1667	ENSDARG00000007377
Regulation of actin cytoskeleton	dre04810	0.2089	ENSDARG00000096549|ENSDARG00000104484
Metabolic pathways	dre01100	0.2142	ENSDARG00000007377|ENSDARG00000074556|ENSDARG00000060554|ENSDARG00000021220|ENSDARG00000071699|ENSDARG00000100786
ECM-receptor interaction	dre04512	0.2249	ENSDARG00000104484
VEGF signaling pathway	dre04370	0.2273	ENSDARG00000096549
Inositol phosphate metabolism	dre00562	0.2297	ENSDARG00000100786

Peroxisome	dre04146	0.2442	ENSDARG00000021220
Salmonella infection	dre05132	0.2583	ENSDARG00000096549
Toll-like receptor signaling pathway	dre04620	0.2676	ENSDARG00000096549
TGF-beta signaling pathway	dre04350	0.2745	ENSDARG00000055283
Phosphatidylinositol signaling system	dre04070	0.2925	ENSDARG00000100786
GnRH signaling pathway	dre04912	0.3252	ENSDARG00000100786
Carbon metabolism	dre01200	0.3273	ENSDARG00000060554
Gap junction	dre04540	0.3316	ENSDARG00000100786
Oocyte meiosis	dre04114	0.3523	ENSDARG00000059131
Cell cycle	dre04110	0.3544	ENSDARG00000042835
Vascular smooth muscle contraction	dre04270	0.3665	ENSDARG00000100786
RNA transport	dre03013	0.3919	ENSDARG00000069951
Cytokine-cytokine receptor interaction	dre04060	0.4071	ENSDARG00000041041
Protein processing in endoplasmic reticulum	dre04141	0.4327	ENSDARG00000024746
Tight junction	dre04530	0.4327	ENSDARG00000014571
Adrenergic signaling in cardiomyocytes	dre04261	0.4691	ENSDARG00000100786
MAPK signaling pathway	dre04010	0.6311	ENSDARG00000096549

*Bolded entry indicates modification selected for in histone immunoprecipitation. The pathways below the highlighted entry were significantly differentially regulated by the modification.
